# Physiology of spontaneous [Ca^2+^]_i_ oscillations in the isolated vasopressin and oxytocin neurones of the rat supraoptic nucleus

**DOI:** 10.1016/j.ceca.2016.04.001

**Published:** 2016-06

**Authors:** Stepan Kortus, Chinnapaiyan Srinivasan, Oksana Forostyak, Yoichi Ueta, Eva Sykova, Alexandr Chvatal, Martin Zapotocky, Alexei Verkhratsky, Govindan Dayanithi

**Affiliations:** a Department of Molecular Neurophysiology, Institute of Experimental Medicine, Czech Academy of Sciences, Videnska 1083, 14220 Prague, Czech Republic; b Institute of Physiology, Czech Academy of Sciences, Videnska 1083, 14220 Prague, Czech Republic; c Institute of Biophysics and Informatics, First Faculty of Medicine, Charles University in Prague, Salmovska 1, 12000 Prague, Czech Republic; d Department of Neuroscience, Charles University, Second Medical Faculty, V Uvalu 84, 15006 Prague, Czech Republic; e Department of Physiology, School of Medicine, University of Occupational and Environmental Health, Kitakyushu 807-8555, Japan; f Department of Neuroscience, Institute of Experimental Medicine, Czech Academy of Sciences, Videnska 1083, 14220 Prague, Czech Republic; g Department of Cellular Neurophysiology, Institute of Experimental Medicine, Czech Academy of Sciences, Videnska 1083, 14220 Prague, Czech Republic; h University of Manchester, School of Biological Sciences, D.4417 Michael Smith Building, Oxford Road, M13 9PT Manchester, United Kingdom; i Achucarro Center for Neuroscience, IKERBASQUE, Basque Foundation for Science, 48011 Bilbao, Spain; j Department of Neurosciences, University of the Basque Country UPV/EHU and CIBERNED, Leioa, Spain; k University of Nizhny Novgorod, Nizhny Novgorod 603022, Russia; l Institut National de la Santé et de la Recherche Médicale, Unité de recherche U1198, Université Montpellier 2, 34095 Montpellier, France; m Ecole Pratique des Hautes Etudes, Sorbonne, 75014 Paris, France

**Keywords:** AVP, arginine vasopressin, OT, oxytocin, MNCs, magnocellular neurosecretory cells, eGFP, enhanced green fluorescence protein, mRFP1, monomeric red fluorescence protein, SON, supraoptic nucleus, [Ca^2+^]_i_, intracellular Ca^2+^ concentration, FFP, Fast Fluorescence Photometer, SD, Standard Deviation, Hypothalamus, Magnocellular neurosecretory cells, Supraoptic nucleus, Vasopressin, Oxytocin, Transgenic rats, Enhanced green fluorescence protein, Monomeric red fluorescence protein, Osmoregulation, Hyper-osmolarity, Hypo-osmolarity, Dehydration, Lactation, Electrical activity, Ca^2+^ oscillations, Fura-2, Fluorescence spectrofluorimetry, Spationtemporal dynamics, Skewness

## Abstract

The magnocellular vasopressin (AVP) and oxytocin (OT) neurones exhibit specific electrophysiological behaviour, synthesise AVP and OT peptides and secrete them into the neurohypophysial system in response to various physiological stimulations. The activity of these neurones is regulated by the very same peptides released either somato-dendritically or when applied to supraoptic nucleus (SON) preparations *in vitro*. The AVP and OT, secreted somato-dendritically (*i.e.* in the SON proper) act through specific autoreceptors, induce distinct Ca^2+^ signals and regulate cellular events. Here, we demonstrate that about 70% of freshly isolated individual SON neurones from the adult non-transgenic or transgenic rats bearing AVP (AVP-eGFP) or OT (OT-mRFP1) markers, produce distinct spontaneous [Ca^2+^]_i_ oscillations. In the neurones identified (through specific fluorescence), about 80% of AVP neurones and about 60% of OT neurones exhibited these oscillations. Exposure to AVP triggered [Ca^2+^]_i_ oscillations in silent AVP neurones, or modified the oscillatory pattern in spontaneously active cells. Hyper- and hypo-osmotic stimuli (325 or 275 mOsmol/l) respectively intensified or inhibited spontaneous [Ca^2+^]_i_ dynamics. In rats dehydrated for 3 or 5 days almost 90% of neurones displayed spontaneous [Ca^2+^]_i_ oscillations. More than 80% of OT-mRFP1 neurones from 3 to 6-day-lactating rats were oscillatory *vs*. about 44% (OT-mRFP1 neurones) in virgins. Together, these results unveil for the first time that both AVP and OT neurones maintain, via Ca^2+^ signals, their remarkable intrinsic *in vivo* physiological properties in an isolated condition.

## Highlights

• Supraoptic fluorescent vasopressin (AVP-eGFP) and oxytocin (OT-mRFP1) neurones exhibit distinct spontaneous [Ca^2+^]_i_ oscillations.
• Vasopressin triggers [Ca^2+^]_i_ oscillations, intensifies existing oscillations, and exceptionally stops oscillations.
• Hyper- or hypo-osmotic stimuli have an intensifying or inhibitory effect on oscillations, respectively.
• Nearly 90% of neurones from 3 or 5-day-dehydrated rats exhibit oscillations.
• More than 80% of OT-mRFP1 neurones from 3 to 6-day-lactating rats are oscillatory *vs*. about 44% in virgins.

## Graphical abstract

Trace (A) shows a typical transient [Ca^2+^]_i_ response induced by 50 mM K^+^ observed in an AVP-eGFP neurone (inset), Trace (B) shows the spontaneous [Ca^2+^]_i_ oscillations observed in an AVP-eGFP neurone (inset). The corresponding changes in the fluorescence are shown in C and D, respectively.

**Figure:**
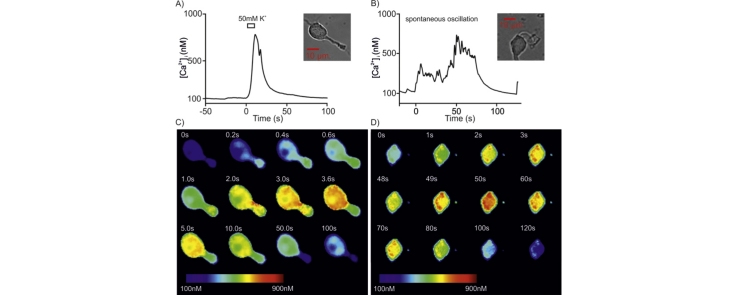

## 1 Introduction

The hypothalamic supraoptic nucleus (SON) contains magnocellular neurosecretory neurones that synthesise the neurohormones arginine vasopressin (AVP) and oxytocin (OT). These hormones are released from axonal projections from these neurones to the neurohypophysis into the portal blood circulation in response to various physiological stimuli such as dehydration, osmotic stimulation, parturition and lactation. Both AVP and OT neurones exhibit characteristic electrical activities. Under physiological settings, the AVP neurones exhibit ‘phasic’ firing activity of action potentials with intervals, whereas OT neurones fire high frequency synchronized bursts of action potentials associated with suckling-induced milk ejection. The characteristic firing patterns, as recorded *in vivo*, are crucial for the efficient release of AVP and OT at the neurohypophysis [1], [2] and from the isolated terminals [3]; and are triggered by Ca^2+^-dependent exocytosis driven by the arrival of action potentials initiated at the cell bodies [4], [5].

In the *in vivo* experiments, the somato-dendritic release of AVP modulates the phasic pattern of AVP neurones electrical activity depending on the initial firing pattern [6]. The intranuclear release of OT, which increases during suckling, increases the excitability of OT neurones [7]. At the subcellular level, AVP induces a transient increase in the intracellular Ca^2+^ concentration ([Ca^2+^]_i_) in isolated AVP-containing neurones [8]. AVP-induced [Ca^2+^]_i_ responses are modulated by specific voltage-gated Ca^2+^ channel subtypes [9] and are mediated by different sub-types of AVP receptors (V_1a_, V_1b_ and V_2_) [6] through activating multiple intracellular transduction signals (PLC and AC) [10]. The autoregulation of AVP and OT neurones is mediated by distinct mechanisms: OT increases [Ca^2+^]_i_ in isolated OT-containing neurones through the activation of specific OT receptors and the release of Ca^2+^ from thapsigargin-sensitive intracellular stores [11], [12], with subsequent activation of store-operated Ca^2+^ entry [13]. In contrast, [Ca^2+^]_i_ responses induced by AVP critically require plasmalemmal Ca^2+^ influx, indicating the primary role for membrane Ca^2+^ channels. Further studies have shown that the existence of a cell specific (AVP and OT) Ca^2+^ homeostasis and Ca^2+^ clearance mechanisms depends upon physiological conditions related to the specific electrical firing patterns of these neurones [14], [15].

The changes in AVP release induced in response to plasma osmolarity fluctuations are mediated by regulation of action potential discharge in the cell bodies [16] and the SON neurones are defined as osmosentive [17], [18], [19]. Other studies have demonstrated that both AVP and OT neurones are activated during chronic dehydration, but with a marked difference in the pattern of their responses [20]. AVP is also shown to be involved in the promotion of water conservation during periods of dehydration [21]. Several previous studies have periodically observed that some isolated AVP and OT neurones (identified by immunocytochemistry or by their [Ca^2+^]_i_ responses to AVP or OT), displayed spontaneous [Ca^2+^]_i_ oscillations under normal experimental conditions [6], [8], [11], [12], [15], [22], [23]. Hitherto, however, neither the identity nor characteristics of these oscillatory neurones, nor the physiological properties of these [Ca^2+^]_i_ oscillations were clearly established.

In the present study, we studied [Ca^2+^]_i_ dynamics in i) AVP-sensitive and OT-sensitive (in terms of their specific [Ca^2+^]_i_ responses) neurones from wild type adult male and virgin Wistar rats; and ii) identified AVP and OT neurones from homozygous transgenic male, as well as from virgin and lactating Wistar rats models expressing (1) an arginine vasopressin (AVP)-enhanced green fluorescent protein (AVP-eGFP) [24]; (2) an oxytocin-monomeric red fluorescent protein 1 (OT-mRFP1) [25] and, (3) in homozygous double transgenic rats simultaneously bearing AVP-eGFP and OT-mRFP1 to visualize both AVP and OT neurones in the same animal [15], [21], [26].

## 2 Materials and methods

### 2.1 Animals and experimental procedures

Adult male Wistar rats (wild type) and three different homozygous transgenic rats were used in this study: transgenic rats expressing an arginine vasopressin-enhanced green fluorescent protein (AVP-eGFP) [25], transgenic male or female or lactating rats expressing an oxytocin-monomeric red fluorescent protein 1 (OT-mRFP1) [25] and double transgenic (expressing both markers) to visualize AVP and OT neurones [15]. A homozygous line was identified among the offspring of two heterozygous parents by finding exclusively heterozygous progeny from the mating of transgenic offspring rat with a wild-type Wistar rat. The double transgenic AVP-eGFP/OT-mRFP line was generated by mating homozygous AVP-eGFP with OT-mRFP transgenic rats. All transgenic rats used in the study were screened by polymerase chain reaction analysis of genomic DNA extracted from rat ear or tail biopsies before breeding and use in the experiments. The PCR was performed using the oligonucleotide primers (AVP-eGFP: sense sequence, 5′-CAC CAT CTT CTT CAA GGA CGA C-3′; antisense sequence, 5′-ATG ATA TAG ACG TTG TGG CTG TTG T-3′; and OT-mRFP: sense sequence, 5′-GTG AAG CAC CCC GCC GAC AT-3′; antisense sequence, 5′- TGC ATT ACG GGG CCG TCG GA-3′). The animals (weighting 150–300 g; 4–8 weeks old) were bred and housed at 22–23° C, 12:12 h light/dark cycle (lights on 07:00–19:00 h), with food and drinking water available ad libitum. The animals were sacrificed by decapitation after anaesthesia with 5% isoflurane for 5 min, the brain was rapidly removed and SON was dissected.

All animals (weighting 150–300 g; 3–8 weeks old; lactating rats weighted about 500 g; 12–16 weeks old) were bred and housed at 22–23° C under normal laboratory conditions (12:12 h light/dark cycle, lights on 07:00–19:00 h) with food and drinking water available *ad libitum*. Transgenic rats were screened by polymerase chain reaction analysis of genomic DNA extracted from rat ear or tail biopsies before breeding and use in the experiments. For each experiment, animals were sacrificed by decapitation after deep anaesthesia with 5% isofluran for 5 min, the brain was rapidly removed and SON was dissected (see below). All experiments were performed in accordance with the European Communities Council Directive of 24 November 1986 (86/609/EEC) regarding the use of animals in research, and were approved by the Ethics Committee of the Institute of Experimental Medicine, Academy of Sciences of the Czech Republic, Prague, Czech Republic (project experiment license #CZ 205/2010 revised in 2013).

### 2.2 Isolation of supraoptic neurones

SON neurones were acutely dissociated by enzymatic and mechanical treatments as described previously [8], [11] with some modifications [14], [27]. In brief, SON tissues (1 mm long, 0.5 mm thick, 0.5 mm wide) were dissected and enzymatically dissociated by incubation for 30 min in oxygenated HEPES-buffered normal Locke’s solution (NL; in mM: 140 NaCl, 5 KCl, 2CaCl_2_, 1 MgCl_2_, 10 glucose, 10HEPES, pH was adjusted to 7.25 with Tris; the osmolarity was 298–300 mOsm/l; temperature 37° C) supplemented with 1 mg/ml deoxyribonuclease I, 0.5 mg/ml proteases X, and 0.5 mg/ml protease XIV. Unless stated otherwise, most of the chemicals were purchased from Sigma-Aldrich (St. Louis, USA). After incubation, tissues were washed with NL and triturated gently using a Gilson-Pipetman (1 ml) with polypropylene white pipette-tip to isolate SON cells. Cells were plated onto glass bottom dishes (22 mm in diameter, 0.17 mm in thick: WillCo Wells-Amsterdam). Unless otherwise stated, all stock solutions of the drugs used in this study were dissolved in this total ion-free distilled H_2_O (EMD Millipore Corporation, Germany). The hypotonic solution was prepared by reducing the appropriate Na^+^ concentration and in the hypertonic solution the osmolarity was increased by adding mannitol.

### 2.3 [Ca^2+^]_i_ measurements using fluorescence photometer system

The [Ca^2+^]_i_ in isolated SON neurones was measured with a fluorescent Ca^2+^ indicator Fura-2 according to the procedure previously described [8] with appropriate revisions [27]. In brief, the SON cells were incubated with 2.5 μM Fura-2 AM (Invitrogen, Carlsbad, CA, USA) with 0.02% Pluronic F-127 (Molecular Probes, Eugene, OR, USA) at 37 °C for 50 min. Two microscope systems were used for [Ca^2+^]_i_ measurements: a fluorescence microspectrofluorimetry system (FFP; Fast Fluorescence Photometer, Zeiss, Jena, Germany) for single-detector experiments and an imaging system using a CCD camera as a detector for video imaging and for measurement of [Ca^2+^]_i_ from neurones obtained from transgenic animals.

### 2.4 [Ca^2+^]_i_ measurements using the FFP system

This system is based on an epi-fluorescence inverted microscope (Axiovert 10, Zeiss, Jena Germany). The excitation light from a Xenon lamp passed through bandpass filters mounted on a computer-controlled rotating wheel that allows alternate stimulation at 340 ± 10 and 380 ± 10 nm with a frequency of 3.3 Hz. The excitation light was deflected by a dichroic mirror (FT 425, Zeiss, Jena, Germany) through an oil-immersion objective (Plan Neofluar 100 × 1.30, ph. 3, Zeiss, Jena, Germany). Fluorescence emission from individual cells was spatially limited by a diaphragm adjusted to the cell size (10–15 μm).

### 2.5 [Ca^2+^]_i_ measurements using CCD video-imaging system

Video imaging of [Ca^2+^]_i_ was performed using an Axio Observer D1 (Zeiss) inverted microscope equipped with filters for monitoring GFP and RFP fluorescence, and epifluorescence oil immersion objectives (Plan Neofluar 100 × 1.30, FLUAR 40X/1.3 oil and FLUOR 20 × 0.75, Zeiss). This allowed us to visualize and identity the SON neurones obtained from AVP-eGFP and OT-mRFP1expressing animals. The excitation light from a Xenon lamp passed through a Lambda D4 ultra-fast wavelength switching system (Sutter Instruments) with a maximum switching frequency of 500 Hz. The fluorescence intensity was detected by using a cooled CCD camera (AxioCam MRm, Zeiss) and the whole system was controlled by Zeiss ZEN Imaging software (2012-SP2/AxioVision SE64 Rel. 4.8.3). The fluorescence intensity was measured with excitations at 340 and 380 nm, and emission at 510 nm.

To estimate [Ca^2+^]_i_ in nM a calibration was performed on a few neurones for both systems. An estimation of [Ca^2+^]_i_ was determined from the f_340_/f_380_ ratio using the Grynkiewicz equation [28]. The calibration parameters for the FFP system were R_min_ = 0.24, R_max_ 4.66, β = 3.39. Calibration performed with the imaging system gave R_min_ = 0.2, R_max_ = 7.2, β = 7.7. The dissociation constant for Fura-2 at 37 °C was assumed as K_D_ = 224 nM.

### 2.6 Drugs application

Solutions were exchanged using a multiple capillary perfusion system, as described previously [29], [30] with appropriate modifications [31]. Briefly, the 200 μm inner diameter capillary tubing was placed close to the tested cell (<500 μm). Solutions were applied through a temperature controlled (set at 37 °C) device, applied through a computer controlled multichannel peristaltic pump (REGLO ICC, Ismatec) using tubing with 0.64 mm inner diameter. Each tubing was fed by a reservoir 30 cm above the bath and connected to a temperature control device (Model: TC-324B; Harvard-Paris, France). The solutions flow rate was set to 500 μl/min for inlet and about 5% slower for outlet. Additional outlet tubing was placed close to the edge of the dish for maintaining 2 mm solution (about 750 μl volume) level in the dish throughout the measurement period. This setup ascertains local perfusion with linear flow without any mechanical disturbance and minimal fluctuation of solution level.

### 2.7 Data pre-processing

Each [Ca^2+^]_i_ trace was first classified as oscillating or non-oscillating. Changes in [Ca^2+^]_i_ with an amplitude of at least 30 nM that occurred within 120 s (or faster) were considered to be “Ca^2+^ events”, and traces or parts of traces in which such events occurred repeatedly were considered “[Ca^2+^]_i_ oscillations”. The threshold of 30 nM was used as a value sufficient for excluding the interference of the noise inherent in the signal. Only [Ca^2+^]_i_ traces with sustained oscillations lasting for at least 10 min were included in further analysis. Traces with strong trend or obvious artefacts were removed from the data set. To reduce noise, all data were filtered by a 2nd-order Butterworth low-pass filter with a cut-off frequency of 0.5 Hz (with filter coefficients estimated by the MATLAB function *butter* and filtration performed by *filtfilt*). MATLAB 2015a and its Statistics, Machine Learning, and Signal Processing toolboxes were employed in all numerical procedures.

Although ratiometric measurements using Fura-2 reduce the effect of photo bleaching, some trend of it exists, especially in longer-lasting experiments. This is because signals from 340 nm and 380 nm do not bleach equally and the ratio is rising even without a change in [Ca^2+^]_i_. We estimated this trend by averaging 55 traces with a duration of more than 15 min and fitting the averaged trace with a linear function of time. This gave a trend expressed as 0.011 nM/s (or; 0.66 nM/min). This increase was then subtracted from all individual traces.

### 2.8 Data analysis and statistical evaluation

**Fig. 1:**
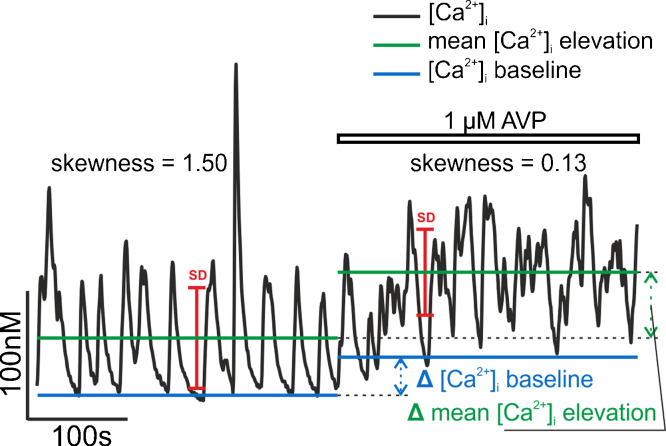
Example of evaluation of [Ca^2+^]_i_ oscillations. For both segments of the recording (300 s in NL buffer and 300 s with added 1 μm AVP), four basic parameters are evaluated: the [Ca^2+^]_i_ baseline, the mean [Ca^2+^]_i_ level, the standard deviation of the trace, and the skewness of the [Ca^2+^]_i_ distribution (Methods, Section 2.8). The difference in parameter values is shown graphically for the [Ca^2+^]_i_ baseline and the mean [Ca^2+^]_i_ level.

Four basic parameters were computed for each individual trace or its segment: the [Ca^2+^]_i_ baseline, the mean [Ca^2+^]_i_ level, the standard deviation of the trace, and the skewness of the [Ca^2+^]_i_ values. These parameters were computed separately for each trace segment corresponding to a defined physiological condition (for example, application of AVP). Only trace segments longer than 300 s were considered. An example of such evaluation is shown in Fig. 1.

The [Ca^2+^]_i_ baseline $c_{\text{B}}$ was estimated as the mean of the 20 smallest [Ca^2+^]_i_ values in each trace or trace segment. The mean [Ca^2+^]_i_ level was calculated as the average of all [Ca^2+^]_i_ values. *i.e.*, $\bar{c}=\left(1/N\right)\left(\overset{N}{\underset{k=1}{\sum }}c_{k}\right)$, where N is the number of [Ca^2+^]_i_ samples in the trace segment and $c_{k}$ are the sample values.

The standard deviation ${\text{SD}}_{\text{Ca}}$ was computed as the square root of the variance of [Ca^2+^]_i_ values within the trace segment. *i.e.*, ${\text{SD}}_{\text{Ca}}=\sqrt{\text{Var}}$, where $\text{Var}=\left(1/N\right)\left(\overset{N}{\underset{k=1}{\sum }}{\left(c_{k}-\bar{c}\right)}^{2}\right)$. The parameter ${\text{SD}}_{\text{Ca}}$ quantifies the spread of the [Ca^2+^]_i_ values within the trace. The asymmetry of this spread is quantified by the distribution skewness γ, defined as

$$\gamma =\frac{\mu_{3}}{{\left({\text{SD}}_{\text{Ca}}\right)}^{3}}\text{,}$$

where $\mu_{3}=\frac{1}{N}\overset{N}{\underset{k=1}{\sum }}{\left(c_{k}-\bar{c}\right)}^{3}$ is the third central moment of the distribution of [Ca^2+^]_i_ values. For a symmetric distribution (such as the normal distribution), γ=0, while for an exponential distribution, γ=2. Positive or negative values of γ indicate a distribution skewed to the right or to the left, respectively (see Section 3.1).

Differences between the trace segments were evaluated based on the four trace parameters defined above. For each parameter, the difference of its value before and after drug application was calculated for each cell trace (see example in Fig. 1). This difference was then averaged over the cells in the given group, and the paired *t*-test was used to determine if the difference was statistically significant. The *p*-values reported in Results represent the significance level for rejecting the null hypothesis of zero difference. The MATLAB function *t-*test was used.

In figures displaying data fitted by a linear approximation, least-squares fitting were performed using the MATLAB function *polyfit*. As a measure of goodness of the linear fit we use the coefficient of determination $R^{2}$ calculated as

$$R^{2}=1-\frac{\sum_{k=1}^{N}{\left(c_{k}-c_{k}^{'}\right)}^{2}}{\sum_{k=1}^{N}{\left(c_{k}-\bar{c}\right)}^{2}}\text{,}$$

where $c_{k}$ denotes the measured [Ca^2+^]_i_ values and $c_{k}^{'}$ the values estimated by the linear approximation.

In all [Ca^2+^]_i_ trace figures, the bars above the trace denote an application of test substances. Unless otherwise indicated specifically, the data are presented as mean values ± SEM (n = the number of observations).

## 3 Results

### 3.1 General features of the calcium oscillations in SON neurones

**Fig. 2:**
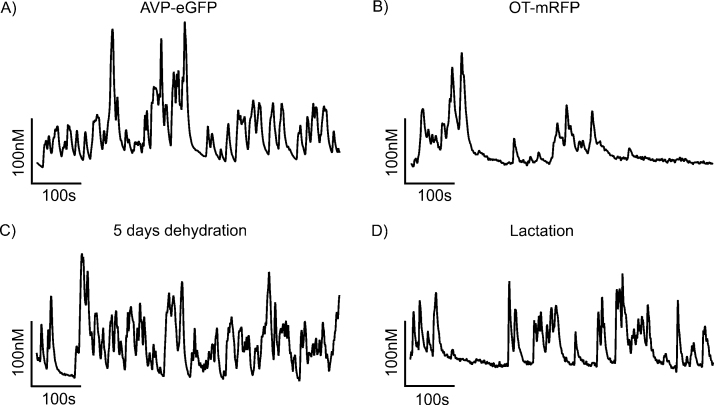
Representative traces of spontaneous [Ca^2+^]_i_ oscillations. Panels A, B: recorded under normal condition in identified OT neurone (A) and AVP neurone (B). Panels C, D: recorded in neurone obtained from 5 days dehydrated rat (C) and from 6-day lactating rat (D).

**Fig. 3:**
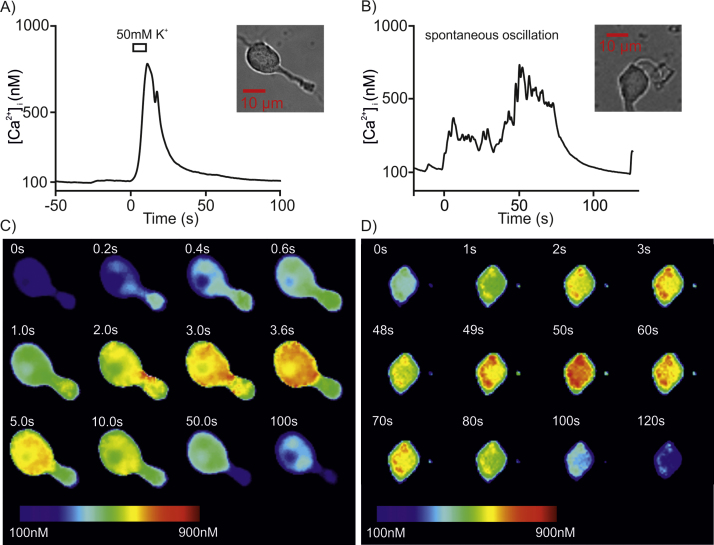
Trace (A) shows a typical transient [Ca^2+^]_i_ response induced by 50 mM K^+^ observed in an AVP-eGFP neurone (inset). Trace (B) shows the spontaneous [Ca^2+^]_i_ oscillations observed in an AVP-eGFP neurone (inset). The corresponding changes in the spatial pattern of fluorescence are shown in C and D, respectively.

We observed spontaneous [Ca^2+^]_i_ oscillations in 79 out of 112 unidentified neurones (71%). Among identified neurons, 12 out of 15 AVP cells (80%) and 9 out of 15 OT cells (60%) exhibited oscillations. Fig. 2 shows a typical example of [Ca^2+^]_i_ oscillation in an identified AVP-eGFP neurone (A) and in OT-mRFP1 neurone (B). To compare the course of spontaneous oscillations *vs*. an evoked transient, we show in Fig. 3A; (video file-1) a typical transient [Ca^2+^]_i_ response induced by 50 mM K^+^ observed in an AVP-eGFP neurone, while Fig. 3B; (video file-2) shows the spontaneous [Ca^2+^]_i_ oscillations observed in an AVP-eGFP neurone. The corresponding spatial changes in the fluorescence are shown in Fig. 3C and D, respectively.

For each spontaneous oscillation trace, we computed the four basic parameters defined in Section 2.8. We then evaluated if these parameter values differed among the three cell groups (unidentified, AVP and OT). No statistically significant difference was found (assessed by unpaired *t*-test). This primarily reflects the large variability among the oscillation traces within each cell group.

**Fig. 4:**
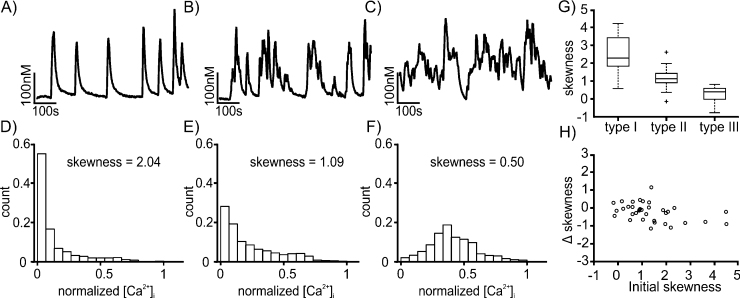
A-C show representative traces from AVP-eGFP neurones in normal condition, and correspond to oscillations of type I, II, and III, respectively (see main text). D-F show the corresponding histograms of [Ca^2+^]_i_ values recorded in the traces located above the histograms. (G) Range of skewness values (showed as standard box plot) in oscillation traces of the indicated type. (H) Control plot showing the difference of skewness in 2nd half and 1st half of [Ca^2+^]_i_ trace recorded in normal conditions (compare to Figs. 5 C, D and 7 C).

The pattern of [Ca^2+^]_i_ oscillations was highly heterogeneous, and can be qualitatively classified into the following three types. The type I pattern is characterized by Ca^2+^ events of regular amplitude and frequency (Fig. 4A); type II consists of irregular events that frequently overlap and form bursts (Fig. 4B); type III contains of oscillations of high frequency, during which [Ca^2+^]_i_ remains continually elevated above the baseline level (Fig. 4C). We visually examined the [Ca^2+^]_i_ traces recorded in 79 unidentified neurones and classified them, based on the qualitative criteria stated above. Type I activity was observed in 29 cells, type II in 34, and type III in 16 cells.

As we observed that the pattern of oscillation often changed when an external stimulus was presented (see following subsections), we sought a simple quantitative classifier that would allow us to systematically express such pattern changes. The frequency or amplitude of the oscillation peaks could not be used for this purpose, as these parameters become ill-defined for irregular oscillations (Fig. 4C). We found, however, that the skewness of the distribution of [Ca^2+^]_i_ values permits distinguishing the basic oscillation patterns. As shown in Fig. 4D–F, the distribution of the [Ca^2+^]_i_ values within the trace has a highly asymmetrical shape in case of the oscillation pattern of Type I (as in Fig. 4A), moderately asymmetrical for the type II pattern (as in Fig. 4B), and symmetrical for the type III pattern (as in Fig. 4C). The strong asymmetry of the distribution in type I arises from the shape of the stereotypical oscillation peaks: a fast rise in [Ca^2+^]_i_ followed by a slower, exponential decay. Correspondingly, the distribution of [Ca^2+^]_i_ values is approximately exponential, with skewness value γ=2.04 in the example in Fig. 4D. In type II, the irregular parts of the oscillation result in a nonexponential tail in the distribution and a lower skewness (γ=1.09, Fig. 4E). Finally in type III, the fast deviations up and down from the mean [Ca^2+^]_i_ level result in an approximately symmetrical distribution with nearly zero skewness (γ=0.5 in Fig. 4F). Fig. 4G shows the range of skewness values in the neurones of each type, where the type was determined by visual examination of the trace as discussed above. As these ranges have low overlap, the skewness can be used as a quantitative classifier, replacing the qualitative visual inspection. In the following, we consider traces with skewness above 1.52 as type I, between 1.52 and 0.53 as type II, and below 0.53 as type III.

### 3.2 Effect of osmolarity on spontaneous [Ca^2+^]_i_ oscillations

**Fig. 5:**
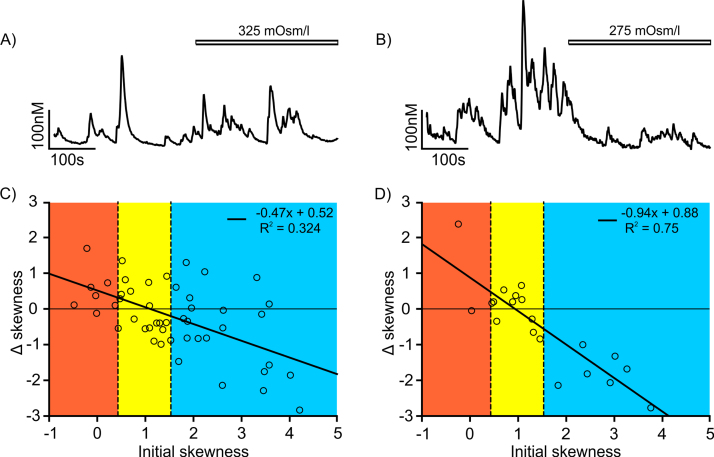
Effect of osmotic change on [Ca^2+^]_i_ oscillations. (A, B): The [Ca^2+^]_i_ trace from neurones subjected to A: hypertonic solution (325 mOsm/l; bar), (B) hypotonic solution (275 mOsm/l; bar). (C, D) Change in skewness resulting from exposure to tonic solution, plotted as function of initial skewness (before exposure). (C) hypertonic, (D) hypotonic solution.

To evaluate the effect of osmolarity, [Ca^2+^]_i_ dynamics in oscillating neurones was monitored for 300 s in NL with a standard osmolarity of 295–300 mOsm/l. Then the perfusion solution was switched to either hypo-osmotic (275 mOsm/l) or hyper-osmotic (325 mOsm/l) solution and [Ca^2+^]_i_ dynamics was monitored for another 300 s. Representative examples of oscillating neurones subjected to hyper- (A) or to hypo- (B) osmotic stimuli are shown in Fig. 5.

**Fig. 6:**
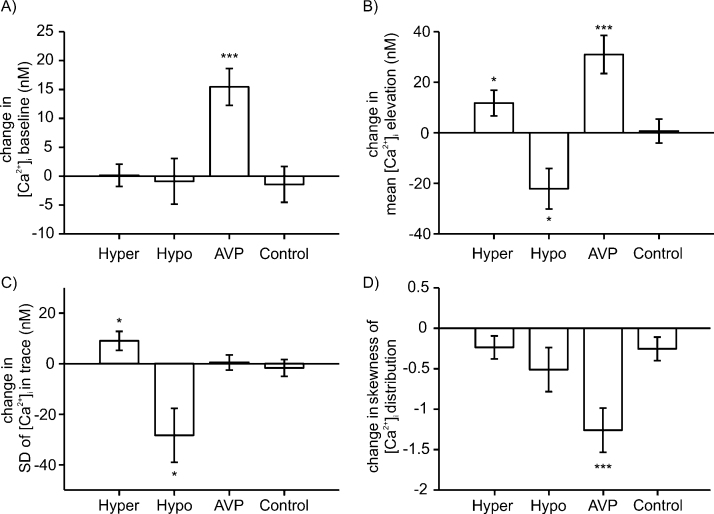
Modulatory effect of AVP and osmolarity on [Ca^2+^]_i_ oscillations. The four panels show, for each evaluated [Ca^2+^]_i_ trace parameter, the change in its value following the application of the specified stimulus (hyper-osmolar, hypo-osmolar, AVP). The respective numbers of measured neurones are: hyper-osmolar, n = 48; hypo-osmolar, n = 20; AVP, n = 31. Panel (A) shows the change in [Ca^2+^]_i_ baseline, (B) in mean [Ca^2+^]_i_ level, (C) in spread (SD) of [Ca^2+^]_i_ within the trace, and (D) in skewness of the [Ca^2+^]_i_ distribution. The bars show the mean value of the parameter change and the respective standard error of the mean. Statistical significance is indicated as: ^*^ for *p*-value < 0.05; ^**^ for p < 0.01, and ^***^ for p< 0.001 (paired *t*-test). In the column marked “Control”, the panels show the differences between the first and second half of a [Ca^2+^]_i_ trace, with the entire trace recorded in normal condition (no stimulus); n = 38. None of these differences were statistically significant.

For each neurone, we calculated the four basic trace parameters, and evaluated if the change in these parameters in normal *vs*. hypo- or hyper osmolarity condition was statistically significant (using the paired *t*-test as described in Section 2.8). The results are summarized in Fig. 6. For neurones exposed to hypo-osmotic solution (n = 20) we recorded a significant decrease in mean [Ca^2+^]_i_ level (22.8 ± 5.0 nM, *p*-value 0.01) and in the spread ${\text{SD}}_{\text{Ca}}$ of [Ca^2+^]_i_ within the trace segment (28.3 ± 8.0 nM, *p*-value 0.02). The other two evaluated parameters, the [Ca^2+^]_i_ baseline and the skewness of the [Ca^2+^]_i_ distribution, did not change significantly (p = 0.82 and 0.08). For neurones exposed to hyper-osmotic solution (n = 48), the increase of mean [Ca^2+^]_i_ level was 11.8 ± 5.1 nM and the increase of ${\text{SD}}_{\text{Ca}}$ was 7.0 ± 3.7 nM. Similarly the hypo-osmotic case, these changes were statistically significant (with *p*-value 0.02), while the changes in baseline and skewness were not (p = 0.1 and 0.9).

Even though the change in skewness was not significant when averaged over all neurones in the group, we did observe significant trends when the initial state of the neurone (*i.e.*, the state in the normal osmotic condition) was taken into account. The skewness of the [Ca^2+^]_i_ distribution tends to increase in neurones that had low initial value of skewness, and to decrease in neurones that had high initial skewness (Fig. 5). This observation is valid for both hyper- (Fig. 5C) and hypo- (Fig. 5D) osmotic stimuli. To summarise, exposure to an osmotic stimulus tends to make [Ca^2+^]_i_ activity more regular, bringing the oscillation into a type II pattern (intermediate range of skewness values, marked in yellow in Fig. 5C, D).

### 3.3 Effect of AVP on [Ca^2+^]_i_ oscillations

**Fig. 7:**
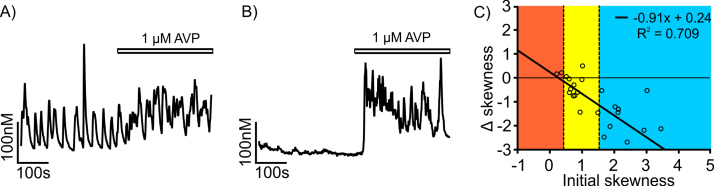
Trace A represents spontaneous Ca^2+^ oscillations observed from SON neurone. The same neurone was subjected to 1 μM AVP (shown in bar). Trace B is an example of a silent and non-oscillating neurone which 1 mM AVP was applied and triggered oscillation. C: Change of the skewness introduced by application of AVP plotted as a function of initial skewness. Coloured areas correspond to oscillatory types (blue—type I, yellow—type II, red—type III) introduced in Fig. 1. Majority of neurones display decrease in skewness corresponding to higher activity. (For interpretation of the references to colour in this figure legend, the reader is referred to the web version of this article.)

We tested the response of 28 oscillating neurones to 1 μM AVP. In each neurone, we compared the oscillations before and after application AVP; the results are summarized in Fig. 6. In majority of neurones the AVP had an obvious enhancing effect on the oscillation (for an example, see Fig. 7A). While SD_Ca_ of [Ca^2+^]_i_ for the trace did not change (Fig. 6C), AVP caused a highly significant increase (p = 0.0003) of the mean [Ca^2+^]_i_ level with average change of 31.0 ± 7.5 nM and a highly significant increase (p = 0.0002) of [Ca^2+^]_i_ baseline with an average change of 13.4 ± 3.2 nM. In addition, AVP elicited very significant (p = 0.001) negative change in the skewness of the [Ca^2+^]_i_ distribution giving a strong average decrease of 1.26 ± 0.27. When the initial skewness was in the intermediate range (type II oscillation), it was only weakly affected by AVP, while in neurones with high initial skewness (type III), AVP typically prompted a switch to type II oscillatory behaviour (Fig. 7C). Beside the effect on oscillating neurones, in some cases (n = 5) AVP triggered oscillations in silent neurone (example in Fig. 7B) and in one case, the AVP attenuated the oscillations (Fig. not shown).

These results mainly indicate a strong enhancing effect of the AVP on [Ca^2+^]_i_ oscillations in magnocellular neurones. These AVP effects on oscillations were mimicked by the specific AVP-V_1a_ receptor agonist [6] and were unaffected by a specific V_1a_ antagonist, SR 49059 [8] (results not shown).

### 3.4 [Ca^2+^]_i_ oscillations persist in neurones obtained from rats subjected to dehydration

Three groups of animals were prepared. In the control group, the rats were maintained under normal conditions (see Section 2) with unlimited access to water. In two other groups, the animals were deprived of water for 3-day and 5-day, respectively. In neurones obtained from 3-day dehydrated rats 18 of 20 cells were spontaneously oscillating. In neurones isolated from 5-day dehydrated rats 9 of 10 neurones were continuously oscillating. The percentages of the spontaneously oscillating neurones form both 3-day and 5-day dehydration animals were 90%, as compared to 71% in the control group of unidentified neurones (Section 3.1). The pattern of oscillations obtained from dehydrated rats was not different from oscillations in normal conditions and none of the four trace parameters exhibited statistically significant differences among these groups (unpaired *t*-test).

### 3.5 Spontaneous [Ca^2+^]_i_ oscillations in neurones from lactating rats

We also measured the [Ca^2+^]_i_ oscillations in identified OT-mRFP1 neurones from 3 to 6-day- lactating rats. The number of oscillating neurones isolated from lactating animals, 18 of 23 (78%), was significantly increased compared to the OT-mRFP1 neurones from transgenic adult virgin rats: 7 of 16 (44%). However, no significant difference was detectable among the patterns of oscillations in these groups of neurones (normal and lactation; Fig. 2A *vs*. D).

## 4 Discussion

In the present study, we report, for the first time, a detailed analysis of the spontaneous [Ca^2+^]_i_ oscillations of SON AVP and OT neurones in isolated condition, and show how these oscillations are affected by the physiological state of the animal (dehydration, lactation) and by exposure to extracellular stimuli (osmotic change, AVP). The majority of AVP neurones (about 80%) and about 60% of OT neurones were oscillatory. Under lactating conditions, there was a significant increase in the number of oscillatory OT neurones (from 44% to about 80%). Spontaneous [Ca^2+^]_i_ oscillations exhibited a wide range of patterns, ranging from regular oscillations with stereotypical [Ca^2+^]_i_ peaks to irregular fast oscillations (Section 3.1). To our knowledge, AVP and OT neurones of the SON represent the exceptional case of defined neuronal sub-populations with distinct electrical activities [1] but with highly heterogeneous patterns of spontaneous [Ca^2+^]_i_ dynamics (present study). Given the heterogeneous character of the oscillations, their consistent characterization by frequency and amplitude was not feasible. Instead, we relied on a combination of four quantitative parameters: the [Ca^2+^]_i_ baseline, the mean [Ca^2+^]_i_ level, the spread (standard deviation) of [Ca^2+^]_i_ values, and the skewness of the [Ca^2+^]_i_ distribution. The skewness parameter, which quantifies the asymmetry of the distribution of [Ca^2+^]_i_ values, was used to classify the oscillation patterns (Section 3.1), and permitted a quantitative evaluation of the change in oscillation character following the presentation of a stimulus (Sections 3.2–3.3).

It has been known that SON neurones are osmosensitive and that their behaviour, notably, the electrical activity of AVP neurones acutely isolated from the rat supraoptic nucleus, is increased by hypertonicity and inhibited by hypotonicity in the absence of neighbouring glial cells and without any synaptic connectivity [32], [33]. Under these conditions, hypertonic stimuli excited the cells by increasing the activity of non-selective cation channels and thus causing membrane depolarisation, whereas hypo-osmotic solutions inhibit AVP neurones through a hyperpolarisation caused by a reduction in the basal activity of the non-selective cation channels. In terms of Ca^2+^ signalling, we report, for the first time, that a vast majority of SON neurones were sensitive to osmotic changes, suggesting that both AVP and OT neurones employ Ca^2+^ signals for osmoregulation. We found that both hypo- and hyper-osmolarity changed the mean [Ca^2+^]_i_, level as well as the amplitude of the variations around this mean (*i.e.*, SD of [Ca^2+^]_i_ in the traces).

The effects of dehydration on AVP and OT neuronal activity, as well as on release of AVP and OT at the level of soma and neurohypophysis have been widely discussed [33], [34], [35], [36]. Of interest, we found that almost all SON neurones (about 90%) became oscillatory during 3–5 days of dehydration, consistent with the need to have more AVP release both at soma and at nerve terminals; and therefore to increase the plasma AVP level to fulfil the physiological demands.

Another aspect of our study was in dissecting various effects of AVP on the spontaneous [Ca^2+^]_i_ oscillations. In previous studies, using *in vivo* electrophysiology and push-pull techniques, the somatodendritic release of AVP was shown to modulate electrical activity of magnocellular neurones [7], [37]. Similar results were obtained in the SON slice preparations [13], [38]; as well as in the *in vitro* release at the level of neurohypophysis or from isolated SON neurones [39]. In addition, it was also demonstrated using SON slice preparations that the electrical activities of AVP neurones show several patterns: (i) regular bursting at regular intervals, (ii) slow-irregular and (iii) fast-continuous (see review of [12], [15]); and are regulated (positive or negative) by the AVP itself [38]. Here we demonstrate that [Ca^2+^]_i_ oscillations observed in AVP neurones similarly display several patterns and AVP modulated these oscillations in a likewise manner: in silent neurones, AVP triggered the oscillations; in oscillating neurones, AVP intensified these oscillations, and in a rare case, AVP attenuated a pronounced oscillation.

The autoregulatory mechanism of OT neurones has been clearly established [11], [12] in terms of Ca^2+^ signals. OT-induced [Ca^2+^]_i_ responses are not only modulated by OT receptors but also OT activates thapsigargin-sensitive intracellular Ca^2+^ stores [13], [15]. In *in vivo* experiments, or in slice preparations, OT always showed an excitatory/positive and synchronised effect on their firing behaviour, leading to the release of a bolus amount of OT to meet their end functions. In our experiments, about 60% of OT neurones showed spontaneous oscillations in [Ca^2+^]_i_ (44% in identified OR-mRFP1 neurones from virgins), these neurones were osmosensitive and sensitive to dehydration. More strikingly, nearly 80% of OT neurones from lactating rats became oscillatory. This finding suggests the possibility, as of *in vivo* effects, that almost all OT neurones should exhibit spontaneous [Ca^2+^]_i_ oscillation, which would facilitate a massive release of OT during lactation *vs*. non-lactating/virgin state or in males. However, further experiments are necessary to identify whether spontaneous [Ca^2+^]_i_ oscillations of all OT-mRFP1 neurones are synchronised during lactation, as well.

## 5 Conclusions

In the present paper we show, for the first time, that fluorescent magnocellular supraoptic AVP-eGFP and OT-mRFP1 neurones exhibit distinct spontaneous [Ca^2+^]_i_ oscillations in an isolated condition under various experimental conditions. These properties are shown to mimic their intrinsic electrical behaviour under physiological conditions such as osmotic shock, dehydration and lactation. The understanding of autoregulatory mechanisms of AVP and OT neurones [12], regulated by peptides they synthesize on their own, is reinforced by the present data on their spontaneous [Ca^2+^]_i_ activity.

## Conflict of interest

The authors state that they have no conflict of interest pertaining to this manuscript.

## Authors’ contribution

SK, CS, OF, GD: performed experiments.

YU, OF: prepared and maintained the heterozygous and homozygous transgenic rats for both AVP and OT and developed double transgenic rats for AVP and OT; performed genotyping, manuscript writing.

SK, MZ, AV, GD: data and statistical methods and analysis; manuscript writing.

AV, GD: prepared the concept of the project.

AC, ES, GD: project management and logistics, manuscript writing.

## Appendix A Supplementary data

Supplementary Video S1 Spatiotemporal dynamics of Ca^2+^ response in an AVP-eGFP neurone. The spatial distribution of [Ca^2+^]i is shown during a typical transient response induced by 50 mM K+. The video sequence is assembled from time lapse images of fluorescence intensity, acquired as described in Section 2.5. The colour scale corresponds to the estimated local [Ca^2+^]i value. The elapsed time is shown at bottom left. The integrated [Ca^2+^]i value is given at bottom left and in the progressively drawn trace, with green bar indicating the time interval during which K+ was applied.

Supplementary Video S2 Spatiotemporal dynamics of spontaneous Ca^2+^ oscillations in an AVP-eGFP neurone. The spatial distribution of [Ca^2+^]i is shown during a spontaneous oscillation in normal condition. The video sequence is assembled from time lapse images of fluorescence intensity, acquired as described in Section 2.5. The colour scale corresponds to the estimated local [Ca^2+^]i value. The elapsed time is shown at bottom left. The integrated [Ca^2+^]i value is given at bottom left and in the progressively drawn trace.
