# Supplementary figures and images for: Physiology of spontaneous [Ca2+]i oscillations in the isolated vasopressin and oxytocin neurones of the rat supraoptic nucleus
Source: Cell Calcium. 2016 Jun;59(6):280–8. doi: 10.1016/j.ceca.2016.04.001 (PMC4969632; doi:10.1016/j.ceca.2016.04.001)

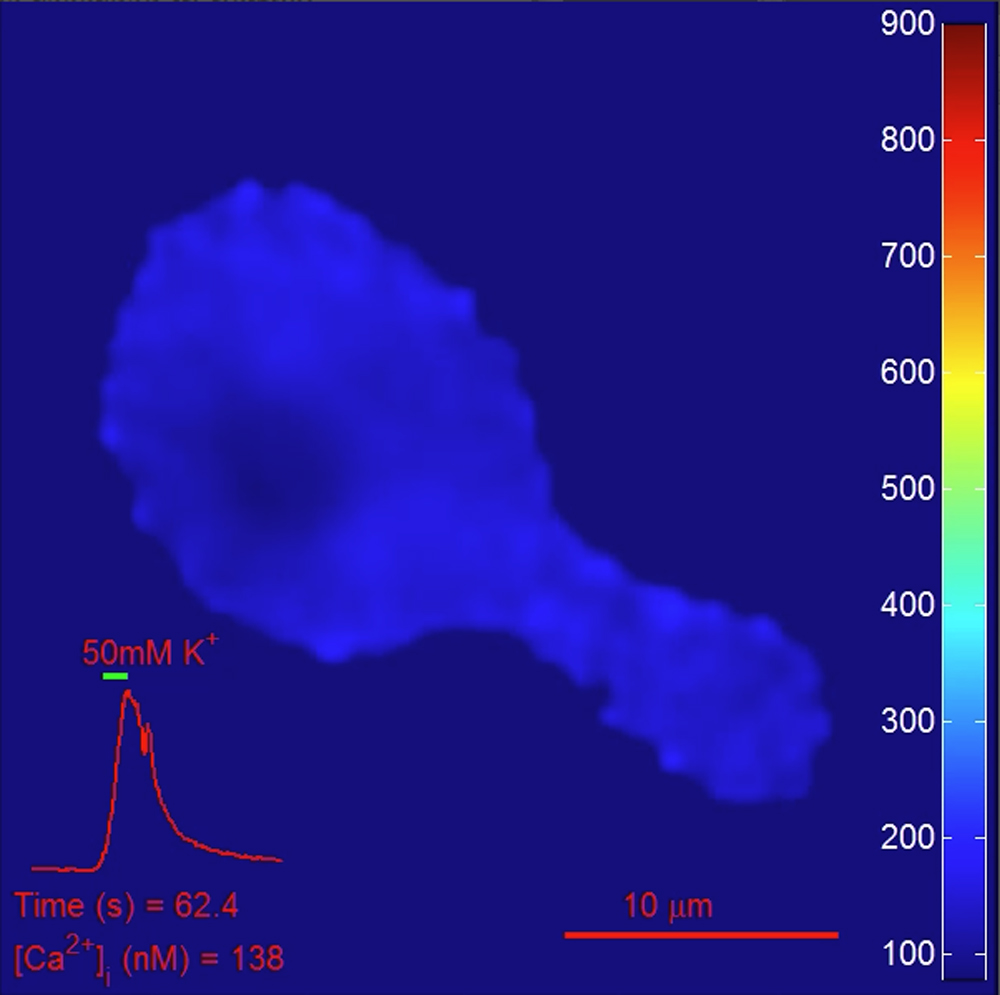

Supplement: Supplementary Video S1 — Spatiotemporal dynamics of Ca2+ response in an AVP-eGFP neurone. The spatial distribution of [Ca2+]i is shown during a typical transient response induced by 50 mM K+. The video sequence is assembled from time lapse images of fluorescence intensity, acquired as described in Section 2.5. The colour scale corresponds to the estimated local [Ca2+]i value. The elapsed time is shown at bottom left. The integrated [Ca2+]i value is given at bottom left and in the progressively drawn trace, with green bar indicating the time interval during which K+ was applied. [file mmc1.jpg]

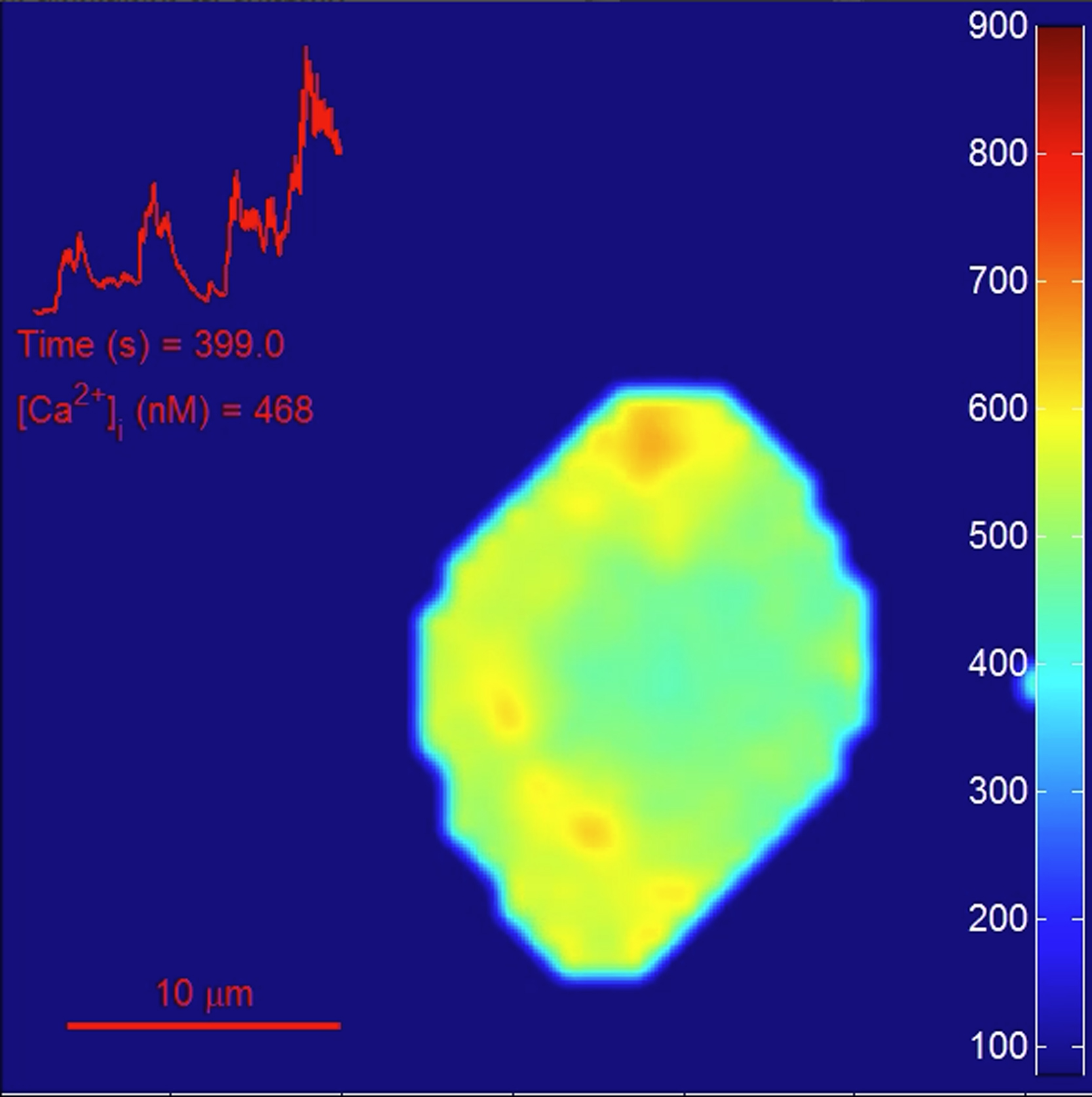

Supplement: Supplementary Video S2 — Spatiotemporal dynamics of spontaneous Ca2+ oscillations in an AVP-eGFP neurone. The spatial distribution of [Ca2+]i is shown during a spontaneous oscillation in normal condition. The video sequence is assembled from time lapse images of fluorescence intensity, acquired as described in Section 2.5. The colour scale corresponds to the estimated local [Ca2+]i value. The elapsed time is shown at bottom left. The integrated [Ca2+]i value is given at bottom left and in the progressively drawn trace. [file mmc2.jpg]
